# Mediterranean natural extracts improved cognitive behavior in zebrafish and healthy rats and ameliorated lps-induced cognitive impairment in a sex dependent manner

**DOI:** 10.1186/s12993-022-00190-8

**Published:** 2022-02-25

**Authors:** Matteo M. Pusceddu, Julia Hernandez-Baixauli, Francesc Puiggrós, Lluis Arola, Antoni Caimari, Josep M. Del Bas, Laura Baselga

**Affiliations:** 1 Eurecat, Centre Tecnològic de Catalunya, Unitat de Nutrició I Salut, Reus, Spain; 2 Departament de Bioquímica I Biotecnologia, Grup de Recerca en Nutrigenòmica, Universitat Rovira I Virgili, Tarragona, Spain

**Keywords:** Cognition, Memory, Learning, Mediterranean Natural extracts, Neuroinflammation

## Abstract

**Background:**

Several findings suggest neuroinflammation as a contributing factor for the onset of psychiatric disorders such as Alzheimer’s disease, depression, and anxiety. There is increasing evidence pointing out that the Mediterranean diet influences brain and behavior. Mediterranean herbs and spices have been shown to be within those components of the Mediterranean diet involved in cognitive enhancement. Thus, we investigated the influence of Mediterranean natural extracts (MNE), Rosemary extract (RE) and *Glycyrrhiza glabra* root extract (GGRE), on cognitive behavior.

**Results:**

Adult zebrafish were exposed to RE or GGRE (100 and 250 mg/L) treatments. Both MNE improved memory retention during the T-maze test, although no improvements were observed during the novel object preference. Similarly, chronic administration of RE (150 mg/Kg) and GGRE (150 mg/Kg) improved, respectively, spatial and retention memory, as assessed by the Morris Water Maze (MWM), and the Elevated Plus Maze (EPM) in healthy male rats. However, no improvements were observed during the novel object recognition. Finally, male, and female rats were chronically treated with lipopolysaccharide [(LPS) 300 ug/kg] and orally administered with RE. Interestingly, RE reversed LPS-induced cognitive deficit during the MWM and EPM in female rats.

**Conclusions:**

We found that MNE improved cognition in both zebrafish and rats. Moreover, MNE rescued LPS-induced cognitive impairment in a gender-specific manner. Therefore, our study supports the view that zebrafish represent a valuable preclinical model for drug discovery in neuroscience. These findings contribute to an exciting and growing body of research suggesting that MNE may play an important role in the prevention of cognitive impairment.

## Background

Chronic inflammation is now considered to be central to the pathogenesis of psychiatric disorders including Alzheimer’s disease, depression, and anxiety. Recent findings have shown that dysregulation of the inflammatory system may lead to memory dysfunction and cognitive impairment both at preclinical [1] and clinical [2] level.

Accumulating translational evidence has proposed that the quality of diet is a crucial and common determinant for mental health [3, 4]. Healthful eating patterns such as the Mediterranean diet, have been shown to offer protective effects on brain function, such as memory and cognitive processes [5, 6]. A growing number of data indicates that native Mediterranean herbs and spices are within those components of the Mediterranean diet involved in memory and cognitive enhancement [7–12].

Liquorice is a root extract from the *Glycyrrhiza glabra* plant that is widely distributed from southern Europe to the northwestern part of China. A number of studies have shown that chronic *Glycyrrhiza glabra* roots extract (GGRE) improves cognition in rodents as assessed by the passive avoidance test, the elevated plus maze (EPM) and the Morris Water Maze (MWM) [9, 13]. GGRE has also been shown to ameliorate cognitive impairment induced by diazepam- scopolamine- and ethanol-induced amnesic effects [9, 14].

*Rosmarinus officinalis* is a perennial herb native to the Mediterranean region. One study showed that chronic administration of rosemary extract (RE) improved cognition in senescence Accelerated Mouse-Prone 8 (SAMP8) mice. Specifically, RE improved cognition as assessed by the T-maze test and the novel object recognition (NOR) [8]. Similarly, RE improved spatial memory during the MWM in repetitive mild Traumatic brain injury (rmTBI) rats [15].

Rodents are an excellent tool for advances towards research in the central nervous system (CNS). However, the high cost of animal housing and the length of the experimental studies [16], critically slow down CNS drug discovery and the identification of novel mechanisms of brain function and dysfunction. Among vertebrates, zebrafish (*Danio rerio*) has revealed as a complementary model to experimental studies in rodents for research in neuroscience [17]. Zebrafish show multiple advantages, including high physiological and genetic homology to mammals, rapid development, ease of genetic and experimental manipulations, sensitivity to cognitive tests as well as cost- and space-effectiveness [18]. Although zebrafish are newcomers to studies of learning and memory, they are capable of performing well in a range of learning tasks such as, avoidance learning [19], alternation spatial memory task [20], associative learning task [21], object recognition [22], and automated learning paradigm [23].

Bacterial lipopolysaccharide (LPS) is a potent activator of the immune system. Several studies have shown that chronic administration of LPS in rodents alters the signaling between the immune system and the brain affecting emotional mood and cognitive behavior [1, 24]. Therefore, LPS represents an excellent murine model of cognitive impairment commonly used to study the effects of neuroinflammation in the development of neuropsychiatric diseases.

Thus, the aim of this study was to investigate the effect of two Mediterranean natural extracts (MNE) in memory and cognition, using a screening system based on zebrafish and testing the most prominent extracts in a murine model of cognitive impairment.

## Materials and methods

### Husbandry

All procedures and protocols involving the care and use of laboratory animals were reviewed and approved by the Animal Ethics Committee of the Technological Unit of Nutrition and Health of EURECAT (Reus, Spain) and the *Generalitat de Catalunya* (DAAM 10,026). All sections of this report adhere to the ARRIVE Guidelines for reporting animal research.

#### Zebrafish

A total of 80 adult (6–8 months old; 3–4 cm long; wild-type) male and female short-finned *Danio rerio* zebrafish (0.4–1 g) of heterogeneous genetic background were obtained from ZF Biolabs (Madrid, Spain). Fish were kept at 28.5 ± 0.5 °C on a 14:10-h light/dark cycles [25] in a standalone aquatic flow-through system (Dohse Aquaristik GmbH & Co. KG, Grafschaft-Gelsdorf, Germany), provided with constant filtration and aeration, at a density of 2 fish per liter. Water used in the system consisted of reverse osmosis water supplemented with 0.8 g/L sea salts (OSMO FIT, Hobby, Germany). Adult fish were fed daily with commercially available flake fish food (Ocean Nutrition, Newark, U.S.A.) and brine shrimps. Fish were acclimated to the aquatic system for 2 weeks before experiment begins. Behavioral tests took place during the light phase between 08:00 and 18:00 h. All efforts were made to minimize the number of animals used and their discomfort. After experiments, zebrafish were euthanized by hypothermia.

#### Rats

Male and female (3 weeks old; 80 g) Sprague–Dawley rats (Envigo RMS Spain, Barcelona, Spain) were maintained in the local animal unit. Food and water were available ad libitum and rats were maintained on a 12:12-h light/dark cycles with temperature at 22 ± 1 °C. Male and female rats were housed in separate animal rooms to avoid hormonal effects on the cognitive tests [26]. A total of 80 rats were single housed in plastic cages with sawdust bedding in an enriched environment with shredded paper and a cardboard roll. Rats were left undisturbed except for routine cage cleaning, twice per week, and body weight were measured weekly until treatments. After experiments, adult rats were fastened for 5 h (from 09.00 to 14.00 h) without restriction of water and killed by decapitation.

### Treatments

#### MNE

GGRE (6.2% glycyrrhizic acid) and RE (6% rosmarinic acid) were obtained by Ebro Regaliz (Barcelona, Spain) and Nutrafur (Murcia, Spain), respectively.

#### Zebrafish

**Fig. 1:**
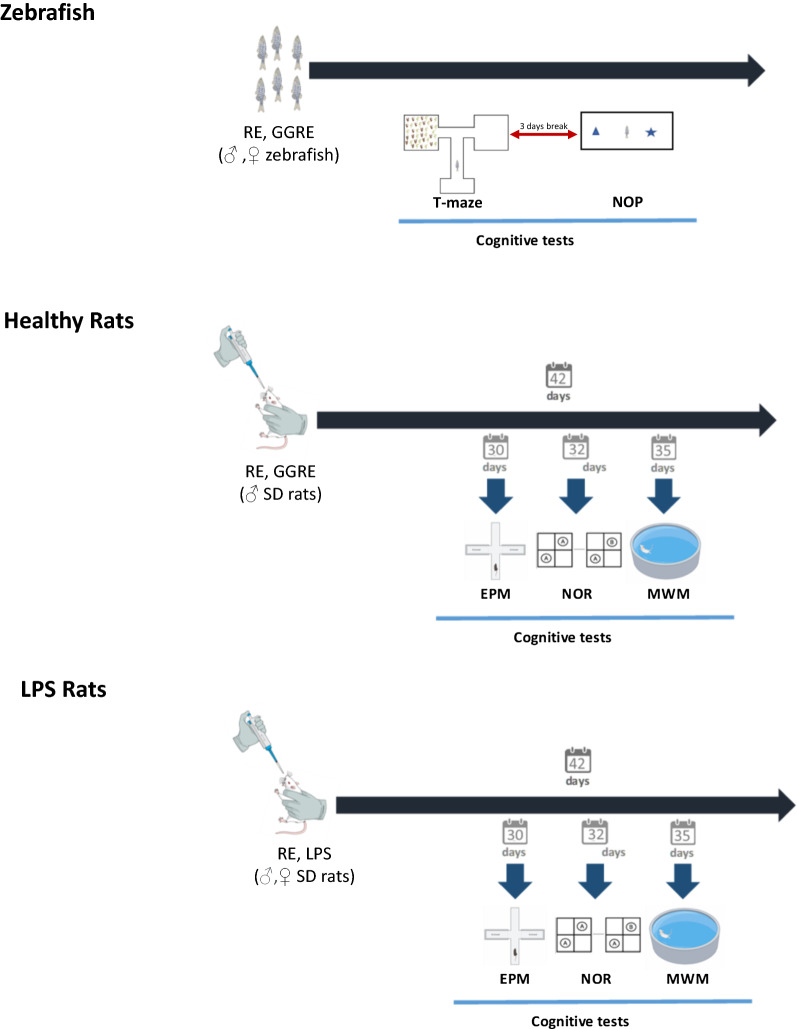
Study design. Experimental timeline showing **A** treatment and animal behavior in zebrafish; **B** treatment and animal behavior in healthy adult male rats; **C** treatment and animal behavior in LPS-treated adult male and female rats

Combined males and females of equal proportion were randomly selected for exposure to GGRE, or RE at a concentration of either 100 or 250 mg/L of water (N = 10/group). Treatments were administered by immersion into a novel water tank for 30 min to minimize stress [27]. After treatment, fish were transferred to another water tank system for 5 min before behavioral tests. To minimize the number of animals used, each fish performed a behavior test battery. Each fish was exposed to the assigned treatment before each behavioral test (twice per fish) (Fig. 1). Control fish were kept under the same conditions used for the treated fish, but without exposure to any treatment.

#### Rats

Two sets of animals were used for two parallel experiments (Fig. 1).

1. *Healthy rats*. Adult male rats were randomly allocated to the following experimental groups: control, GGRE (150 mg/Kg) or RE (150 mg/Kg), (n = 10/ group).
2. *LPS-treated rats*. Adult male and female rats were randomly allocated to the following experimental groups: control, LPS, LPS + RE (n = 10/ group).

Once animals were four weeks old underwent oral administration of either GGRE (150 mg/Kg) or RE (150 mg/Kg) for six weeks. Treatments were dissolved in low-fat condensed milk:water (1:10 v/v) and administered by micropipette. Controls were treated with low-fat condensed milk:water (1:10 v/v), only. As for the LPS-treated rats’ study, LPS (300 ug/kg, i.p.) was administered for seven days, during the fourth week of the study, 2 h after RE or vehicle treatments, when animals were seven weeks old. Controls for the LPS group were i.p. injected with 0.9% sterile saline water for the same period. All treatments were administered every day, between 09.00 and 10.00 h. Animals underwent behavioral testing immediately after LPS treatment (Fig. 1). GGRE and RE treatments were based on previous studies where similar doses were applied [41, 42].

### Behavioral testing

#### Zebrafish

Zebrafish underwent a behavior test battery spread over 5 days: T-maze on days one and two; the novel object preference (NOP) on day five. All behavioral tests were performed in the morning between 9.00 and 12.00 h.

#### T-maze

**Fig. 2:**
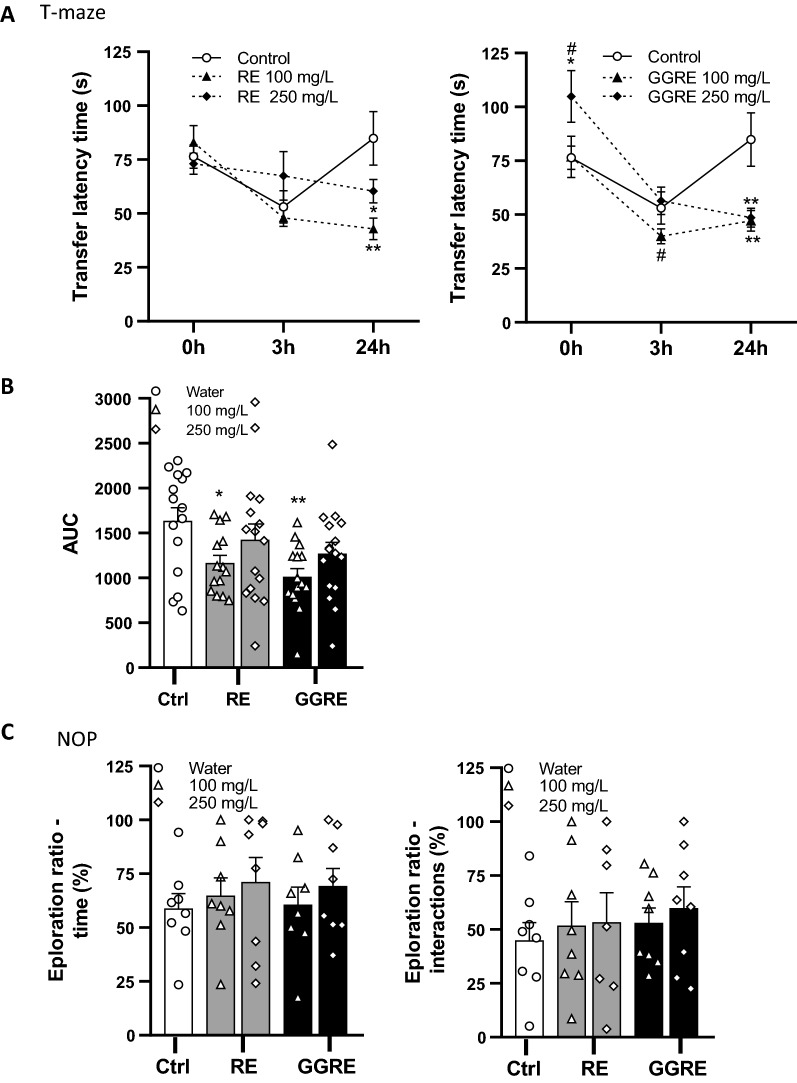
Mediterranean natural extracts improved memory retention **in** zebrafish. **A** Time curve representing TLT during TMT1, TMT2 and TMT3 of the T-maze test; **B** AUC representing TLT during the T-maze test; **C** Time and interaction scores during the NOP task expressed as exploration ratio (%). Data are presented as the mean ± SEM. (∗ *P* < 0.05; ∗  ∗ *P* < 0.01 compared to control; # *P* < 0.05 compared to 250 mg/L)

T-maze was used to measure spatial memory as described previously [28]. The apparatus consisted of a white plexiglass maze with a T shape, filled with 10 cm of tank water. The long arm (50 × 10 cm) was intersected at one of its ends by a short arm (50 × 10 cm). One of the ends of the short arm opens to a larger reservoir (30 cm square) 5 cm deeper than the rest of the maze. The reservoir was enriched with artificial plants and color stones to offer a favorable habitat to the fish. By nature zebrafish would spend more time in the reservoir than in the rest of the maze [28]. The other end of the long arm was connected to a starting zone (30 × 10 cm) by a white removable door (Fig. 2). The protocol consists in three trials [T-Maze T1 (TMT1), TMT2 and TMT3]. During each trial, fish were individually placed in the starting zone for 1 min with the door closed. Then, the start box was raised and lowered after the fish exited. During the TMT1, fish were given a maximum of 4 min to fully explore the maze and encounter the reservoir where they were left for at least 20 s before the end of the trial. Each fish repeated the trial 3 h (TMT2) and 24 h (TMT3) later. After each trial, fish were returned to their home tank. All trials were video-recorded, and the time needed to find the reservoir [transfer latency time (TLT)] was quantified using a digital tracking system (ANY-Maze, version 4.82).

#### NOP

NOP was performed as previously described [29]. Briefly, zebrafish were subjected to 5 min of acclimation to the novel tank (29 × 14 × 18 cm tank filled with 14 L of tank water) followed by 10 min of habituation to two identical objects (training phase). After a recovery period of 10 min, one of the two original objects was randomly replaced with a novel object and interactions were monitored for an additional 10 min (testing phase). To avoid thigmotaxis influence, the objects were placed one in front of the other in an equal distance between the center of the tank and the walls. An 8.4 cm × 8.4 cm box was superimposed over each object and the time the fish spent within the boxes was recorded and scored automatically (ANY-Maze, version 4.82).

#### Rats

Behavioral tests were carried out during the fifth [EPM, NOR and open field (OF)] and sixth (MWM) week of MNE administration, when the animals were eight and nine weeks of age, respectively (Fig. 1). Before each behavioral test, rats were habituated to the test room for 30 min. All experiments were carried out between 9:00 am and 2:00 pm.

#### EPM

EPM was performed to assess retention memory as described previously [30] with some modification. The maze consisted of two open arms (50 × 10 com) and two enclosed arms (50 × 10 × 40 cm) that all extended from a common central platform (10 × 10 cm). The apparatus was raised 60 cm above the floor on a central pedestal. On the training phase, rats were placed individually at the end of the open arm, facing it away from the central platform. The time that the animal took to move from the open arm to one of the two enclosed arms (4 paws in) was recorded. The same procedure was repeated 60 min (acquisition) and 24 h (retention) after training. To become acquainted with the EPM, if the rat failed to enter the enclosed arm within 120 s, it was guided to one of them and let it to explore the maze for further 60 s before to be returned to its home cage (training phase only). Animal behavior was recorded and TLT (acquisition and retention phases) was analyzed using a tracking system (ANY-Maze, version 4.82). The apparatus was wiped clean with 70% ethanol before testing each animal.

#### NOR

NOR was used to assess recognition memory as described previously [31] with some modifications. Briefly, rats were placed in a grey plywood rectangular box (70 × 45 × 45 cm) for 5 min for habituation to the novel arena. The day after, rats were habituated to two identical objects [(A), acquisition phase)] placed in the back left and right corners of the experimental box for 3 min. After a delay of 1 h, one of the two familiar objects was replaced with a novel object (B) and rats were replaced in the middle of the box at the mid-point of the wall opposite the sample objects for a total time of 3 min (retention phase). Box and objects were cleaned with alcohol 70% to avoid any cue smell between each trial. Direct contacts with the objects, include any contact with mouth, nose or paw or minimal defined distance (< 2 cm), were recorded and scored automatically (ANY-Maze, version 4.82). Memory discrimination index (MDI), [(B exploration time – A exploration time)/ (B exploration time + A exploration time)], was used to express recognition memory in rats [32].

#### OF

To assess anxiety-like behavior rats were individually placed in a wooden grey box (70 × 45 × 45 cm) for 5 min. A central area (20 × 40 cm) was drawn in the floor of the apparatus to score time and number of entries in the inner zone. Distance moved, velocity, percentage of time spent in inner zone and frequency of inner zone entries were recorded and analyzed using a tracking system (ANY-Maze, version 4.82). The apparatus was wiped clean with 70% ethanol before testing each animal.

#### MWM

The MWM is a test of spatial learning and reference memory for rodents that relies on distal cues to navigate from start locations around the perimeter of an open swimming arena to locate a submerged escape platform [33]. The swimming arena consisted of a circular metal pool (150 cm diameter × 58 cm tall) filled with tap water (23-25ºC, 40-cm deep). The maze was divided geographically into four quadrants: Northeast (NE), Northwest (NW), Southeast (SE) and Southwest (SW), and four starting positions, North (N), East (E), West (W), South (S), were located at equal distances along the pool rim. A circular white platform (10 cm diameter) was placed in the center of the NW quadrant and submerged 1.5 cm below the water surface. Water was white colored with a non-toxic white paint to hide the platform. Distal cues were arranged around the maze to provide landmarks that the animals could use to navigate to the platform.

#### Acquisition training

Rats underwent five days of training that consisted of four trials/day. At the beginning of each trial rats were individually placed in one of the starting positions (N, E, S, or W) facing the wall of the tank and allowed to explore the maze for 60 s. A different starting position was used for each of the 4 trials on a given day arranged in a semi-random pattern. Once rats reached the platform, they were held there for 20 s. If the platform was not found within the given time, rats were gently assisted to the platform by the experimenter and detained there for 20 s.

#### Removal phase

On day six (removal) the platform was removed, and the rats were allowed to freely explore the maze for 60 s. Rats were individually placed into the quadrant diagonally opposite to the one originally hosting the platform. The amount of time spent in the quadrant where the platform was previously hidden was video recorded and automatically analyzed using a tracking system (ANY-Maze, version 4.82).

### Statistical analysis

All the data are expressed as mean ± standard error of the mean (SEM). Repeated measure (RM)-ANOVA was performed to analyze differences over time among treatments, whereas ANOVAs analysis followed by LSD’s *post-hoc* test was performed to analyze single-time points differences between treatment groups. Student’s *t*-test was used for single statistical comparisons. The level of statistical significance was set at bilateral 5% (*p* < 0.05). All statistical analysis was performed with the SPSS Statistics 26 software (SPSS, Inc., Chicago, IL, United States).

## Results

### Mediterranean natural extracts improved memory retention in zebrafish

#### T-maze

The T-maze was used to assess memory retention in zebrafish. For RE effects on zebrafish, RM-ANOVA revealed an overall learning improvement over time (*f*_(2,76)_ = 6.928, *p* = 0.002) and a significant time by treatment interaction (*f*_(4,76)_ = 4.513, *p* = 0.003), (Fig. 2A). Interestingly, significant time by treatment interaction was mainly due to the treatment effect lasting for 24 h. In contrast, in the control groups cognitive abilities returned to basal at 24 h time point. Accordingly, LSD’s multiple comparisons post hoc analysis showed that RE supplementation reduced TLT of 50% (100 mg/L) and of 30% (250 mg/L) at 24 h time point, compared to controls (Fig. 2A). For GGRE effects on zebrafish, RM-ANOVA revealed an overall learning improvement over time (*f*_(4,74)_ = 15.087, *p* = 0.000) and a significant time by treatment interaction (*f*_(4,74)_ = 4.385, *p* = 0.00), (Fig. 2A). Similar to the RE effects, significant time by treatment interaction was mainly due to the long lasting effects of GGRE up to 24 h. Post hoc analysis showed that TLT was higher in zebrafish treated with GGRE (250 mg/L) at baseline compared to both controls and GGRE 100 mg/L-treated zebrafish. GGRE (100 mg/L) reduced TLT of 25% at 3 h time point compared to controls. Moreover, GGRE, at both concentrations, reduced TLT of 57% at 24 h time point compared to controls (Fig. 2A). Similarly, AUC analysis confirmed the improved learning in both RE- and GGRE-treated zebrafish (1-way ANOVA: *f*_(4,75)_ = 3.092, *p* = 0.021; Fig. 2B). Moreover, post hoc analysis revealed that both RE and GGRE improved memory retention at a concentration of 100 mg/L, only (Fig. 2A).

#### NOP

The NOP task was used to assess recognition memory in zebrafish. All the experimental groups successfully performed the training phase showing no preference for neither one of the familiar objects (data not shown). When zebrafish were exposed to the novel and the familiar objects (test phase) all the groups were able to discriminate the familiar object from the novel one (more than 50%), (Fig. 2C). However, MNE did not induce cognitive improvement during the NOP task.

### Mediterranean natural extracts improved learning and cognition in adult rats

To confirm the findings obtained from the zebrafish study, cognitive behavior was assessed in healthy male rats chronically treated with either RE or GGRE.

#### MWM

**Fig. 3:**
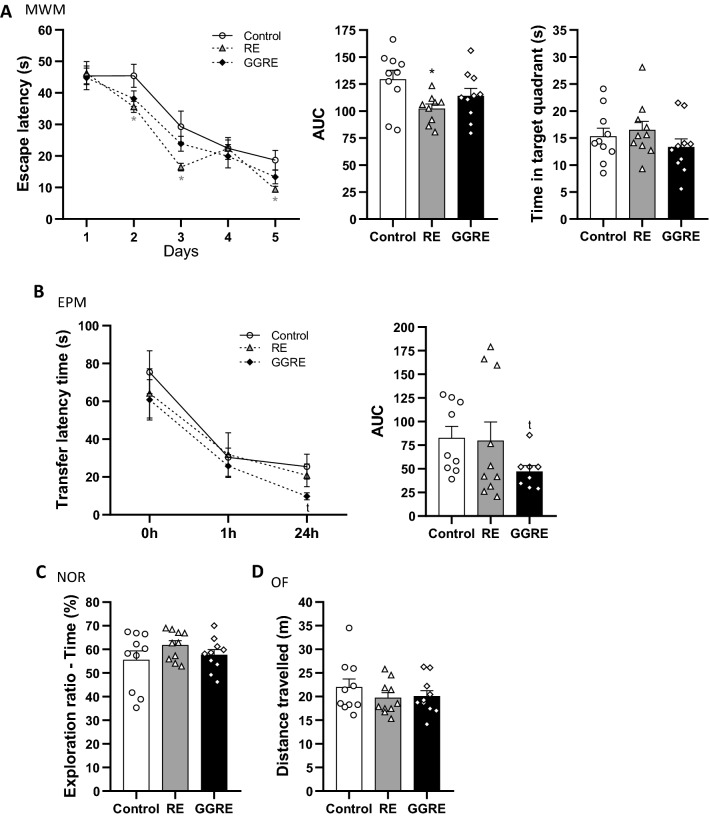
Mediterranean natural extracts improved learning and cognition in adult rats. **A** MWM represented by latency time to reach the platform over 5 training days, AUC and removal phase 24 h after the last training day; **B** EPM represented by the time to reach the reservoir at 0 h (training), 1 h (acquisition) and 24 h (retention) as well as AUC; **C** NOR represented by the time spent with the objects expressed as exploration ratio (%); **D** distance travelled during the OF test. Data are presented as the mean ± SEM. (∗ *P* < 0.05 compared to control; t *P* < 0.05 compared to control, unpaired student’s *t*-test)

To assess whether MNE improved spatial learning and memory, rats were trained over five consecutive days to find a hidden platform in the MWM. RM-ANOVA revealed a significant effect of time (*f*_(4,92)_ = 53.013, *p* = 0.000; Fig. 3A), only. LSD’s multiple comparisons post hoc analysis showed that RE supplementation improved learning at days 2 (*p* = 0.036), 3 (*p* = 0.018) and 5 (*p* = 0.030) of the training sessions compared to controls (Fig. 3A). Similarly, AUC analysis confirmed the improved learning in RE-treated rats (1-way ANOVA: *f*_(2,26)_ = 3.668, *p* = 0.040; LSD’s post hoc test:* p* = 0.012; Fig. 3A) compared to controls. However, no improvements were observed in rats treated with GGRE (Fig. 3A). To assess spatial long-term memory, a probe trial was performed on day 6. However, no improvement induced by MNE was found (*f*_(2,27)_ = 1.522, *p* = 0.236; Fig. 3A).

#### EPM

We further tested the effect of MNE on retention memory by assessing the EPM test. RM-ANOVA revealed that all experimental groups improved TLT towards one of the enclosed arms of the maze over time (*f*_(2,46)_ = 51.430, *p* = 0.000; Fig. 3B). Moreover, single statistical comparison analysis revealed that GGRE-treated rats improved TLT at 24 h’ time point, when compared to controls (student’s t-test: *p* = 0.045; Fig. 3B). Similarly, AUC analysis confirmed memory improvements in GGRE-treated rats (student’s t-test: *p* = 0.027; Fig. 3B). No improvements induced by RE were observed.

#### NOR

Next, we tested whether MNE supplementation can modulate recognition memory as assessed by the NOR task. All experimental groups successfully discriminate the novel object from the familiar one as expressed by an exploration ratio score higher than 50% (Fig. 3C). However, neither RE nor GGRE improved recognition memory compared to controls.

#### OF

None of the treatments altered locomotor activity as assessed by the OF test (Fig. 3D). No other changes such as time and number of crossings in the inner zone were found (data not shown).

### RE reversed LPS-induced cognitive impairment in rats in a sex dependent manner

The study on healthy rats revealed a stronger effect of RE on learning skills and spatial memory compared to GGRE. In accordance, we selected RE to further investigate whether MNE exert any protective effect in a rat model of cognitive impairment induced by neuroinflammation. To this aim, cognitive deficit was induced by chronic injection of LPS (300 mg/kg) for 7 consecutively days to both male and female rats before animal behavior commenced.

#### MWM

**Fig. 4:**
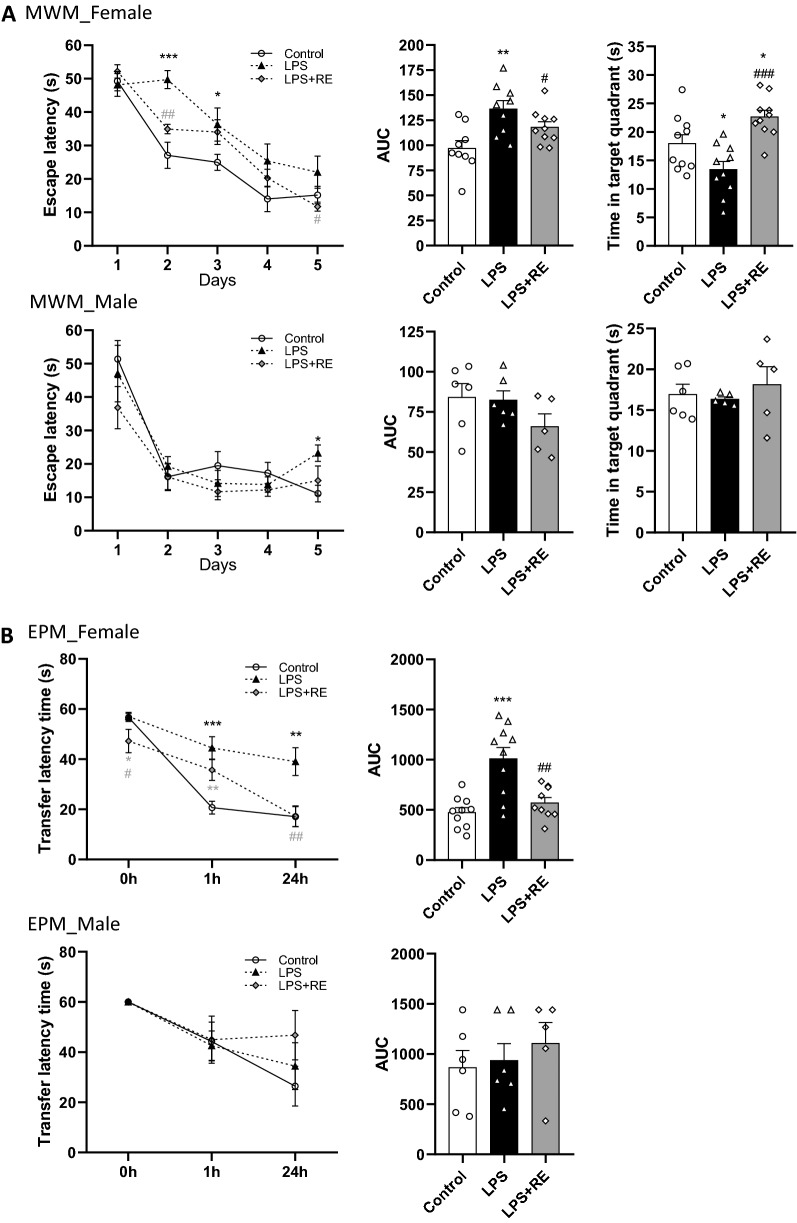
RE reversed LPS-induced cognitive impairment in rats in a sex dependent manner. **A** MWM represented by latency time to reach the platform over 5 training days, AUC and removal phase 24 h after the last training day; **B** EPM score represented by the time to reach the reservoir at 0 h (training), 1 h (acquisition) and 24 h (retention) as well as AUC. Data are presented as the mean ± SEM. (∗ *P* < 0.05; ∗  ∗ *P* < 0.01; ∗  ∗  ∗ *P* < 0.001 compared to control, # *P* < 0.05 ## *P* < 0.01 compared to LPS)

The effects of chronic administration of RE against LPS-induced cognitive impairment during the MWM are shown in Fig. 4A. For RE effects on female rats, RM-ANOVA revealed a significant effect of time (*f*_(4,96)_ = 56.788, *p* = 0.000), a significant time by RE interaction (*f*_(4,96)_ = 4.078, *p* = 0.004) and a significant time by LPS interaction (*f*_(4,96)_ = 2.772, *p* = 0.031), (Fig. 4A). LSD’s multiple comparisons post hoc analysis showed escape latency time to be higher in the LPS group at days 2 (*p* = 0.000) and 3 (*p* = 0.049) of the training phase (Fig. 4A). Interestingly, LPS + RE female rats were able to find the hidden platform faster than the LPS-treated group both at days 2 (*p* = 0.049) and 5 (*p* = 0.049), (Fig. 4A). To assess spatial long-term memory, a probe trial was performed on day 6. One-way ANOVA revealed a significant effect of group on time spent in the target quadrant (*f*_(2,27)_ = 11.348, *p* = 0.000; Fig. 4A). LSD’s multiple comparisons post hoc analysis showed spatial long-term memory deficit in LPS treated rats when compared to controls (*p* = 0.026; Fig. 4A). Interestingly, RE treatment reversed LPS-induced cognitive impairment (*p* = 0.000; Fig. 4A). Moreover, RE + LPS treated rats spent more time in the target quadrant compared to controls (*p* = 0.023; Fig. 4A). Similarly, AUC analysis confirmed that RE reversed LPS-induced learning impairment in female rats (*f*_(2,26)_ = 7.084, *p* = 0.004; Fig. 4A). For RE effects on male rats, RM-ANOVA revealed a significant effect of time (*f*_(4,56)_ = 25.109, *p* = 0.000; Fig. 4A), only. Moreover, LSD’s multiple comparisons post hoc analysis revealed learning deficit at day 5 of the training sessions induced by LPS administration to male rats (*p* = 0.0322; Fig. 4A). However, no further changes were observed in male rats, as referred to AUC analysis and the total time in the target quadrant (Fig. 4A). Therefore, data indicated that RE protective effects in response to LPS stimulation were more pronounced in female than male rats.

#### EPM

The effects of chronic administration of RE against LPS-induced cognitive impairment during the EPM are shown in Fig. 4A. For RE effects on female rats, RM-ANOVA revealed a significant effect of time (*f*_(2,54)_ = 69.210, *p* = 0.000), a significant time by LPS interaction (*f*_(2,54)_ = 8.170, *p* = 0.001), (Fig. 4A). LSD’s multiple comparisons post hoc analysis showed that LPS induced cognitive retention impairment in female rats at both 1 (*p* = 0.000) and 24 h (*p* = 0.002), (Fig. 4B). Interestingly, TLT score was lower in LPS + RE female rats compared to the LPS-treated (*p* = 0.030; Fig. 4B). Moreover, RE reversed LPS-induced memory deficit at 24 h’ time point (*p* = 0.002; Fig. 4B). AUC analysis confirmed the protective effect exerted by RE against LPS-induced cognitive deficit as shown during the EPM test (*f*_(2,26)_ = 13.679, *p* = 0.000; Fig. 4B). No RE effects were observed in male rats (Fig. 4B).

## Discussion

The present studies demonstrate that selective MNE can induce cognitive enhancement in adult zebrafish. Moreover, we showed that chronic administration of both GGRE and RE had similar cognitive improvements in healthy rats. Additionally, we demonstrated that LPS-induced cognitive impairment was reversed by chronic RE administration in female rats only. Our findings provide additional support for MNE as potential memory enhancement as well as potential complementary compounds to current therapeutic interventions in the treatment of cognitive impairment and suggests that MNE may be more efficacious in females with a neuroinflammatory profile.

Rodents have been traditionally used to advance our knowledge on human behavior, such as learning and cognition. However, expanding the range of experimental domains with the use of additional models, is now considered as an important strategy for translational neuroscience research [34]. Recent data suggest zebrafish as an emerging successful model to study cognitive phenotypes and CNS related drug discovery [35–37]. Accordingly, our behavioral results show that both GGRE and RE improved retention memory as indicated by the T-maze test. Similarly, RE has been shown to improve cognitive function in a zebrafish model of amnesia induced by scopolamine [38]. We also found that improvement in retention memory was best at the lowest concentration used (100 mg/L). This suggests that at high doses (250 mg/L) both GGRE and RE may have off-target activity. Ceiling effects have been previously observed with other natural extracts in rats [39, 40]. Interestingly, the results obtained in zebrafish correlated with those observed in healthy male rats. Specifically, RE demonstrated improvements in spatial learning as indicated by the MWM. Moreover, a better recall memory was observed in GGRE treated rats at 24 h, but not 1 h, from the training phase of the EPM task. In a similar study, GGRE improved memory functions in a mice model of LPS-induced neuroinflammation [41]. Moreover, RE has been shown to rescue learning and spatial memory deficits in a rat model of epilepsy induced by treatment with kainic acid [42]. Interestingly, a randomized clinical trial observed that RE treatment positively affected memory performance, anxiety, depression, and sleep quality in 34 Iranian university students [43]. On the other hand, no improvements were found in recognition memory either in zebrafish or in rats as assessed by the NOP and the NOR tests, respectively. There is a wide agreement that both spatial learning and long-term memory are largely dependent on the integrity of the hippocampus [44, 45], whereas its role in recognition memory is controversial. A number of studies suggest no effect of hippocampal lesion in recognition memory [46, 47], while other studies reported significant impairments [48, 49]. Moreover, it is suggested that tasks of recognition memory are mainly supported by cortical structures adjacent to the hippocampus and that are less dependent to hippocampal malfunctioning [50, 51]. For instance, the hippocampus receives projections from the adjacent perirhinal and entorhinal cortices which have been shown to severely disrupt recognition memory in rodents [50]. Thus, our data may suggest that the observed improvements in spatial learning and long-term memory induced by GGRE and RE may be due to their action on neuronal circuitries specifically involved in the regulation of hippocampal-dependent tasks both in zebrafish and rats. However, it should be mentioned that improvements in recognition memory induced by RE and GGRE have been previously reported in mice [41, 52]. The discrepancy with our findings may be found in the applied animal model of cognitive impairment and the type of rodents used. Further analysis is warranted to shed more light on this issue.

Growing evidence suggests that females are more susceptible to the development of mood and cognitive deficits compared to males, leading to a higher risk for psychopathologies [53]. Neuroinflammation has been indicated as one of the main factors that contribute to sex differences in psychiatric diseases such as Alzheimer’s disease, depression and anxiety [54, 55]. LPS injections represent a widely used animal model of neuroinflammation that has been shown to disrupt memory and cognition in rodents [1, 56]. We observed that, at the chosen dose of 300 ug/kg, LPS chronic administration impaired females’ learning, and cognition of a greater extent compared to male rats. Interestingly, RE chronic treatment demonstrated protective cognitive effects in response to LPS chronic injection. However, such effect was blunted by LPS administration in male rats. Such discrepancy may be found on the different immunological response between genders that in turn may be associated with the gender-specific outcomes from RE treatment. Further research is warranted.

In conclusion, we demonstrated that zebrafish can be considered as a valuable preclinical model for drug discovery in neuroscience. The results obtained in zebrafish are, to a great extent, comparable to those observed in healthy rats. Moreover, we provided further evidence that neuroinflammation is involved in the regulation of learning and cognition using a rat model of LPS-induced memory impairment. Notably, the protective effects of the selected MNE against LPS-induced memory impairment were gender-specific. Finally, this study shed light on the beneficial role of MNE administration in enhancing cognitive abilities both in healthy rodents and rats exhibiting cognitive impairment.

## Data Availability

We will make our data available should it be required.

## Abbreviations

CNS: Central nervous system
E: East
EPM: Elevated plus maze
GGRE: Glycyrrhiza glabra roots extract
LPS: Lipopolysaccharide
MDI: Memory discrimination index
MNE: Mediterranean natural extracts
MWM: Morris Water Maze
N: North
NE: Northeast
NOP: Novel object preference
NOR: Novel object recognition
NW: Northwest
OF: Open field
RE: Rosemary extract
RM: Repeated measure
rmTBI: Repetitive mild traumatic brain injury
S: South
SAMP8: Senescence Accelerated Mouse-Prone 8
SE: Southeast
SEM: Standard error of the mean
SW: Southwest
TLT: Transfer latency time
TMT: T-Maze Trial
W: West
